# Tetracycline removal from wastewater via g-C_3_N_4_ loaded RSM-CCD-optimised hybrid photocatalytic membrane reactor

**DOI:** 10.1038/s41598-024-51847-5

**Published:** 2024-01-12

**Authors:** Milad Esfandiaribayat, Mojtaba Binazadeh, Samad Sabbaghi, Milad Mohammadi, Samaneh Ghaedi, Hamid Rajabi

**Affiliations:** 1 Department of Chemical Engineering, School of Chemical and Petroleum Engineering, Shiraz University, Shiraz, Iran; 2 Department of Civil and Environmental Engineering, University of Alberta, Alberta, T6G 2W2 Canada; 3 Department of Nano-Chemical Engineering, Faculty of Advanced Technologies, Shiraz University, Shiraz, Iran; 4 School of Engineering, the University of Manchester, Manchester, M13 9PL UK; 5 Department of Civil and Environmental Engineering, School of Engineering, University of Liverpool, Harrison Hughes Building, Liverpool, L69 3GH UK

**Keywords:** Chemical engineering, Photocatalysis

## Abstract

In this study, a split-type photocatalytic membrane reactor (PMR), incorporating suspended graphitic carbon nitride (g-C3N4) as photocatalyst and a layered polymeric composite (using polyamide, polyethersulfone and polysulfone polymers) as a membrane was fabricated to remove tetracycline (TC) from aqueous solutions as the world's second most used and discharged antibiotic in wastewater. The photocatalyst was synthesised from melamine by ultrasonic-assisted thermal polymerisation method and, along with the membrane, was characterised using various methods, including Brunauer–Emmett–Teller analysis (BET), Fourier-transform infrared spectroscopy (FTIR), X-ray diffraction analysis (XRD), Field emission scanning electron microscopy (FESEM), and Ultraviolet–visible spectroscopy (UV–Vis). The PMR process was optimised, using Design-Expert software for tetracycline removal in terms of UV irradiation time, pH, photocatalyst loading, tetracycline concentration, and membrane separation iteration. It was revealed that a membrane-integrated reactor as a sustainable system could effectively produce clean water by simultaneous removal of tetracycline and photocatalyst from aqueous solution. The maximum removal of 94.8% was obtained at the tetracycline concentration of 22.16 ppm, pH of 9.78 with 0.56 g/L of photocatalyst in the irradiation time of 113.77 min after six times of passing membrane. The PMR system showed reasonable reusability by about a 25.8% drop in TC removal efficiency after seven cycles at optimal conditions. The outcomes demonstrate the promising performance of the proposed PMR system in tetracycline removal from water and suggest that it can be scaled as an effective approach for a sustainable supply of antibiotic-free clean water.

## Introduction

As a pressing concern, water contamination by antibiotics has detrimental impacts on human health and ecosystems, fuelling the proliferation of antibiotic-resistant bacteria and ecological imbalance^1^. Pharmaceutical industries^2^, healthcare facilities^3^, agricultural practices^4^, and wastewater treatment systems^5,6^ are known as major contributors to waterborne antibiotic pollution. Water treatment plants are now grappling with an overwhelming influx of antibiotics in water bodies, exacerbated by population growth and disease outbreaks, thereby necessitating the development of sustainable methods to ensure antibiotic-free water^5^. Tetracycline holds a distinction among antibiotic compounds as the world's second most-used antibiotic, making it widely discharged in wastewater^7^. The chemical stability, solubility, mobility, degradation resistance, and low-concentration activity of tetracycline in water have made its selective removal a challenge, requiring the use of sensitive techniques for its detection and capture^1^. Various treatment methods, such as adsorption, coagulation, chemical precipitation, ion exchange, biodegradation, ozonation, etc., have been widely employed to eliminate pharmaceutical contaminants from water^8^. Nevertheless, the generation of harmful by-products, poor biodegradability and stability, and high operational and maintenance costs hinder their widespread industrial application^9,10^. Consequently, the development of robust technologies is essential to effectively break down these pollutants into non-toxic compounds before their release into the environment^11,12^. A few innovative technologies, such as advanced oxidation processes (AOPs) in^13^ and membrane separation techniques in^14^, are now at the forefront of exploration for proposing antibiotic-free clean water due to their operational efficiency and adaptability.

Photocatalytic membrane reactors (PMRs), combining photocatalysis and membrane technology, is an emerging AOP technology to simultaneously carry out chemical reactions and separation which can be used for removing organic pollutants from water^15–18^. The PMRs initial idea was to overcome the challenge of post-photocatalysis collection of suspended photocatalysts, especially at nanometres dimensions, from water using membranes, also offering higher selectivity and efficiency (enhanced reaction rates, reduced catalyst loss, and simplified separation), long-term stability and eco-friendly operation^6,19,20^. PMRs can be divided into two general types based on the way that photocatalysts and membranes are structurally incorporated (suspended or immobilised). Suspended PMRs can be further categorised into three groups: slurry PMRs with an external membrane module, slurry PMRs with a submerged membrane module (external UV lamps), and slurry PMRs with an immersed membrane module and light source^21^. However, immobilised PMRs can perform enhanced reactivity, better selectivity, and simple catalyst recovery for capturing organic compounds; they may be susceptible to catalyst detachment and membrane fouling^22^. Membrane-immobilized PMRs can also improve mass transfer and stability in the longer run but with limited catalyst loading and potential membrane degradation^23^. Suspended PMRs, on the other hand, can offer efficient catalyst utilisation and anti-fouling properties but are prone to the separation of suspended catalyst particles in severe operating conditions^24^. Hollow fibre and tubular PMRs with better porosity and selectivity, efficient separation and light utilisation may suffer from fouling and scalability limitations. The design of an effective PMR method for purification purposes is mainly controlled by the intended outcomes and trade-offs between reaction rate, selectivity, mass transfer efficiency, catalyst stability, and operational ease^22^. However, PMRs have been reported as a promising approach for capturing organic compounds from water; their sustainable applications for antibiotic removal face a few challenges of antibiotic-specific removal mechanisms, optimal synergic effect, impact of water matrix and coexisting compounds, limited stability and fouling resistance, and scale-up challenges^25^. Further studies are needed to offer innovative formulations for PMRs with efficient synergic effects between photocatalytic reactions and membrane-based separation to fill the gaps for sustainable performance in real applications.

Graphitic carbon nitride (g-C_3_N_4_) as a layered polymeric and metal-free semiconductor is an appealing photocatalyst to be incorporated in PMRs with advantages of nontoxicity, affordability, separability, production simplicity, chemical and thermal stability, excellent visible light absorption, and suitable band structure for photocatalysis^26^. However, it has been extensively studied for applications such as organic pollutant degradation, water disinfection, hydrogen production, and environmental remediation; there are still challenges and opportunities for further exploration to enhance the photocatalytic performance of g-C_3_N_4_ through modifications, heteroatom doping, and hybridisation with other materials^27,28^. In this study, for the first time in the removal of tetracycline (TC) from aqueous solutions, a novel formulation for split-type PMR is developed, utilising nanosheets of g-C_3_N_4_ and a commercial type of polyethersulfone (PES)/polystyrene (PS)/polyamide (PA) membranes. The synthesised PMRs were characterised using BET, TEM, FTIR, XRD, FESEM, EDS, AFM and UV–Vis in TC removal. A systematic experimental approach is employed to investigate the impact of key parameters in PMR-based removal of TC from water, including irradiation duration, pH level, catalyst amount, TC concentration, and filtration iterations. To ensure a robust optimisation of multiple-parameter experimental design and analysis, the combination of the Response Surface Method (RSM) and Central Composite Design (CCD) is also used. The reusability of g-C_3_N_4_/PES/PS/PA-based PMR is also monitored to test the promising stability of the proposed formulation for practical applications of TC removal from waterways. Supplementary information S1 is also provided separately for further details wherever needed.

## Materials and methods

### Materials

Melamine (2,4,6-Triamino-1,3,5-triazine, 99%), hydrochloric acid (HCl, 37%), sodium hydroxide (NaOH, 97%) and ethanol (C_2_H_5_OH, > 99.8%) were purchased from Merck Co. Tetracycline hydrochloride (C_22_H_24_N_2_O_8_·HCl, <  = 100) was obtained from Sigma-Aldrich Co. The double distilled water (DDW) was a product of Zolal Iran Co. (Tehran, Iran). All the chemicals were used as received without any further purifications. To prepare TC stock solution (50 mg/L), 10 mg of TC powder was dissolved in 200 mL of DDW and stirred at 200 rpm for 60 min. The solution was then filtered and stored in a sealed, non-transparent container as stock to prepare TC solutions with concentrations of 10, 15, 20, 25, and 30 mg/L.

### *Synthesis of g-C*_*3*_*N*_*4*_

For g-C_3_N_4_ synthesis, melamine was initially subjected to direct furnace heating. In a pot, 5g of melamine was placed and securely sealed with thick aluminium foil. The furnace temperature was then gradually increased at 10 °C/min up to 520 °C and maintained constant for two h. The resulting yellowish mass (g-C_3_N_4_) was grounded using a mortar. The sieved materials were immersed in DDW, ultrasonicated for 90 min in three stages (to prevent agglomeration, particle stability, and increase specific surface area), and oven-dried at 70 °C for 24 h, as also confirmed by^29^. The resulting g-C_3_N_4_ were kept in a sealed container for characterisation and testing purposes.

### Characterization techniques

Field Emission Scanning Electron Microscope (FE-SEM, MIRA3 TESCAN-XMU, Czech Republic) was utilised for morphological assessment of the synthesised g-C_3_N_4_ and used membrane, at a beam energy level of 30.00 kV under high vacuum conditions. The porous structure of the used materials was analysed based on the Brunauer Emmett Teller (BET) method by the use of the Micromeritics apparatus (ASAP2020, USA). The material's crystallinity was investigated using an X-ray diffractometer (XRD, PANalytical X’Pert Pro-MPD Powder Diffractometer, UK), by which the data were collected in the 2θ range of 5–70°. Fourier-transform infrared spectrometry (FTIR, Bruker TENSOR II, USA) was used to record the surface functional group spectra of the materials over 400–4000 cm^−1^. g-C_3_N_4_ surface charge was evaluated by a Zeta-potential device (Photon Correlation Spectroscopy (VASCO), France). The hydrophilicity of the membrane was studied by the contact angle device (Contact Angle CAG-20) along with the AFM device (PK NanoWizard ULTRA Speed 2), which also determined the surface roughness and morphology of the membrane. UV-DRS device (JASCO V-730) was employed to perform Ultraviolet–Visible spectrophotometry (UV–Vis), obtaining diffuse reflectance spectrum (DRS) and band gap energy in the wavelength range of 200–400nm. The calibration curve for UV absorption by the TC solution can be found in Fig. S1 in the supplementary data.

### Experimental testing and optimisation

**Figure 1:**
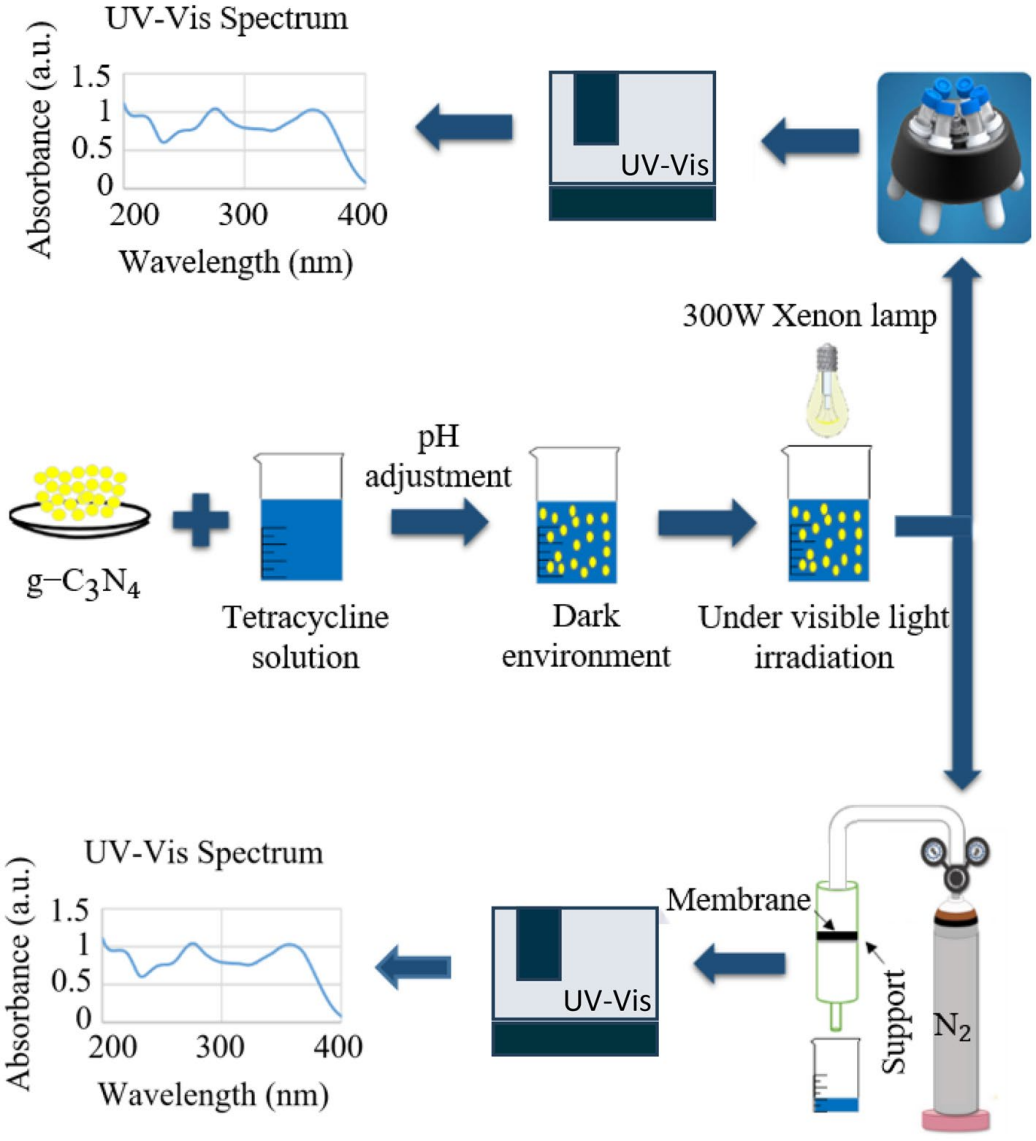
Schematic diagram of a laboratory scale split-type PMR with suspended photocatalyst.

The schematic diagram of the bespoke laboratory-scale slurry PMR system developed for this study is presented in Fig. 1 and Fig. S1. The membrane with a layered structure made of polyester, polysulfone, and polyamide was purchased from Sepanta Polymer Sharif Co. (Tehran, Iran). Table S1 presents the main properties of the commercial membrane. In each test, a predetermined amount of g-C_3_N_4_ (0.2–1 g/L) was dispersed in a solution with specific pH and TC concentrations. Before being exposed to irradiation, the sample was magnetically stirred for 30min in a dark environment to establish an adsorption–desorption equilibrium. Photocatalytic degradation tests were carried out under visible light irradiation for a specific time (60-120min) using a 300W Xenon lamp (TACPRO, WLY202009) with a 420nm cut-off filter. The solution was centrifuged at 7500rpm for 8 min to take photocatalyst-free samples. A cylindrical module with a 5-bar retentate static N_2_ pressure was used to perform the membrane separation process. The UV–Vis spectrophotometer was employed to detect TC concentration in the permeate solution at $\lambda_{\max }$ = 356.5 nm and determine TC removal (%) using the following equation:

1 $$ {\text{Removal efficiency }}\left( {\text{\% }} \right) = \frac{{{\text{C}}_{{0}} - {\text{ C}}_{{\text{t}}} }}{{{\text{C}}_{{0}} }} \times {100} $$

where ${\text{C}}_{0}$ and ${\text{C}}_{\text{t}}$ are initial and final TC concentrations, respectively.

The response surface methodology (RSM), based on a central composite design (CCD), was used in this study for optimisation purposes, using design-expert software (Ver. 12.0.3, USA). The main objective was to analyse the effects of intended independent parameters, develop regression models, and determine the optimal conditions for PMR-based removal of TC from water. The experimental designs were randomised, and mean values were used. The ranges for the independent factors determined based on the existing literature, preliminary tests, and material properties were as follows: irradiation time (60–120 min), initial pH level (7–13), catalyst concentration (0.2–1 g/L), initial TC concentration (10–30 mg/L), and filtration cycles (2–6 times). In total, 50 tests were carried out, and a summary of experimental conditions and their corresponding responses is provided in Table 1 for photocatalytic degradation and PMR removal. The relationship between the independent variables and responses was analysed via fitting a quadratic polynomial function. The significance and precision of the model were then evaluated through variance analysis (ANOVA), and the fitness of the model was expressed using the coefficients of determination (R^2^, R^2^_adj,_ and R^2^_pred_).

**Table 1:** ANOVA results of quadratic model for TC Removal.

Source	Sum of squares	df	Mean square	F-value	P-value	Comment
Model	4008.07	13	308.31	205.52	< 0.0001	Significant
A-Irradiation time	176.40	1	176.40	117.59	< 0.0001	
B-pH	176.40	1	176.40	117.59	< 0.0001	
C-Cat. dosage	57.60	1	57.60	38.40	< 0.0001	
D-TC initial concentration	739.60	1	739.60	493.02	< 0.0001	
E-Number of passes	960.40	1	960.40	640.21	< 0.0001	
BC	8.00	1	8.00	5.33	0.0268	
BD	6.13	1	6.13	4.08	0.0508	
BE	6.13	1	6.13	4.08	0.0508	
A^2^	14.05	1	14.05	9.36	0.0042	
B^2^	658.85	1	658.85	439.19	< 0.0001	
C^2^	372.64	1	372.64	248.41	< 0.0001	
D^2^	812.05	1	812.05	541.31	< 0.0001	
E^2^	19.85	1	19.85	13.23	0.0009	
Residual	54.01	36	1.50			
Lack of fit	45.13	29	1.56	1.23	0.4174	Not significant
Pure error	8.88	7	1.27			
Cor total	4062.08	49				

## Results and discussion

### XRD and FTIR analysis

**Figure 2:**
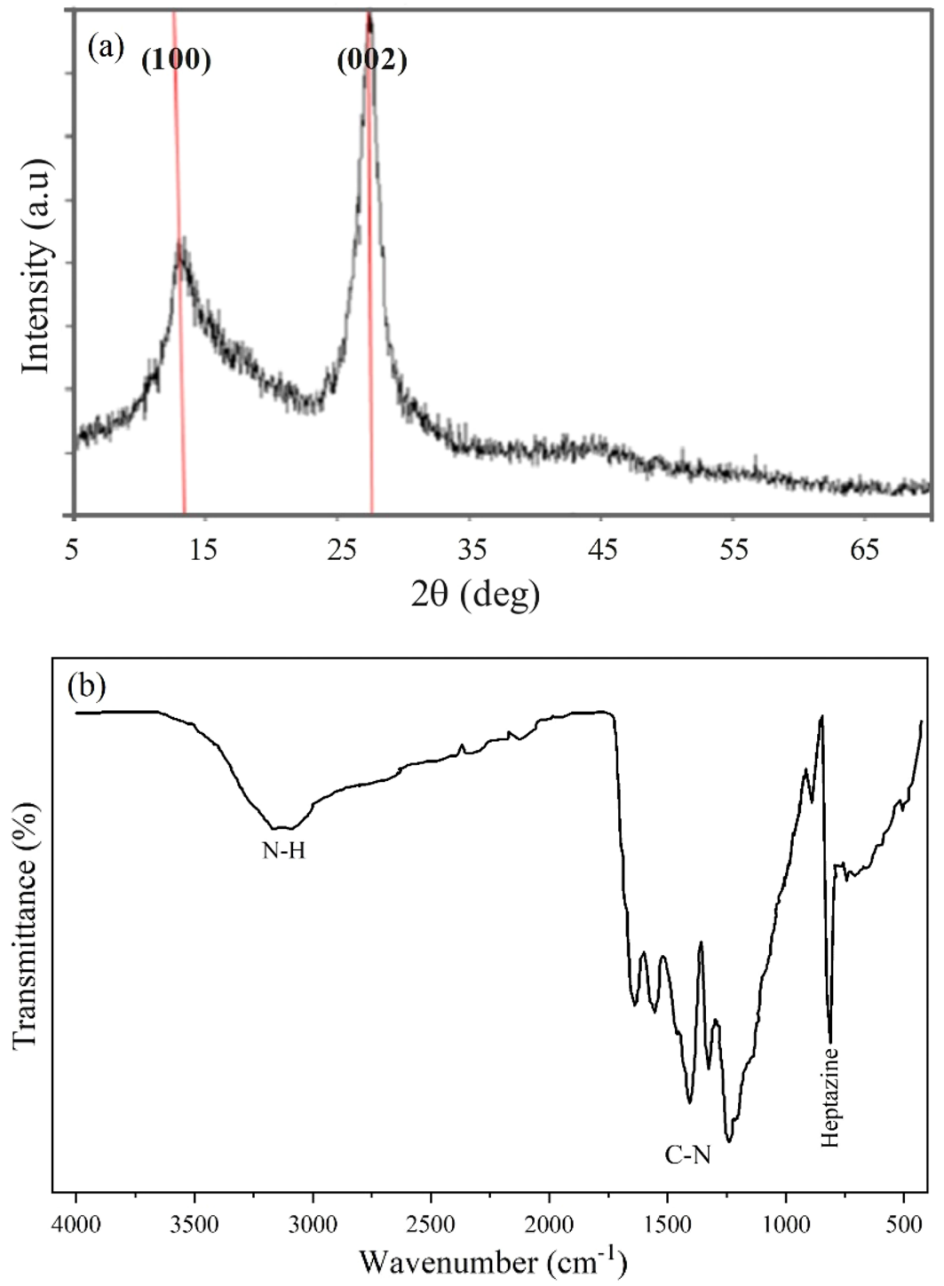
(**a**) XRD pattern and (b) FT-IR absorption spectrum of synthesized $\text{g} - {\text{C}}_{3}{\text{N}}_{4}$.

The g-C_3_N_4_ nanosheets’ XRD patterns are depicted in Fig. 2a. The peaks at 27.66° and 13° can be associated with the (002) and (100) crystal planes of the g-C_3_N_4_ structure, respectively^30^. The sharp peak at 27.66° may indicate the interlayer distance of the g-C_3_N_4_ nanosheets, also known as graphitic carbon nitride, which can be influenced by intercalation, doping, or modification of g-C_3_N_4_. The presence of the (100) crystal plane can confirm that the synthesised materials is in the form of nanoplates rather than bulk material^31^. The (100) plane corresponds to a parallel layer arrangement held together by weak van der Waals forces^32^. The obtained X-ray diffraction spectrum aligns with findings reported in other studies^33–36^. Figure 2b presents the FTIR spectrum of the synthesised g-C_3_N_4_ materials. The wide peak at 3152 cm^−1^ can be related to the N–H stretching of s-triazine rings^37^, while, the peaks ranging 1628–1231 cm^−1^ are attributed to the stretching vibrations of the aromatic C-N heterocycle^38^. The sharp peak at 804 cm^−1^ points to the presence of 3D heptazine structures within the g-C_3_N_4_ nanosheets^33,38,39^.

### FE-SEM, BET and zeta potential analysis

**Figure 3:**
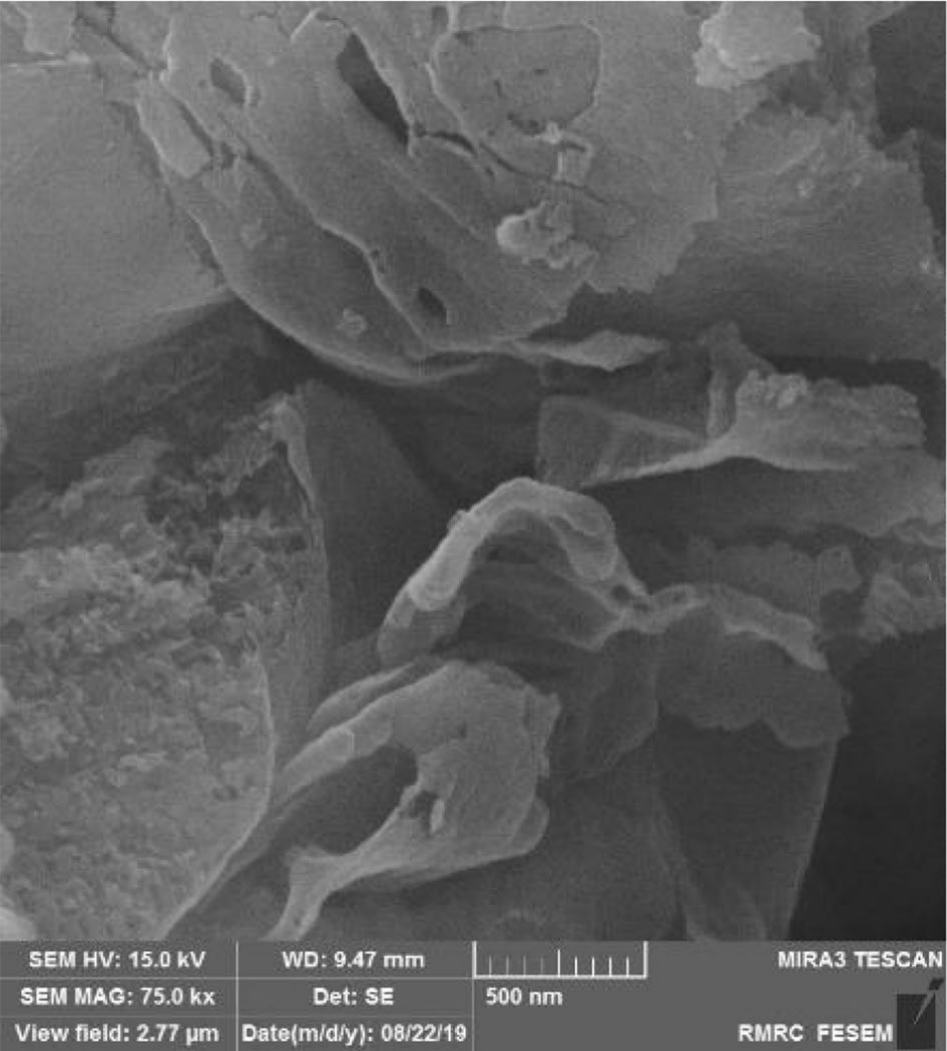
FE-SEM image of synthesised $\text{g} - {\text{C}}_{3}{\text{N}}_{4}$.

The morphology and nanostructured skeleton of the synthesised g-C_3_N_4_ were studied through a FE-SEM analysis and presented in Fig. 3. The FESEM image can provide further evidence of the two-dimensional layered structure of g-C_3_N_4_, appearing as sheets with wrinkles. It can be attributed to the polycondensation of melamine molecules, leading to the formation of g-C_3_N_4_ sheets. The nanostructures of g-C_3_N_4_ tend to have a sheet-like appearance due to their graphite-like structure. Moreover, the FESEM image demonstrated the presence of a porous g-C3N4 structure, with numerous pores observed on the surface as well as within the nanostructure itself.

**Figure 4:**
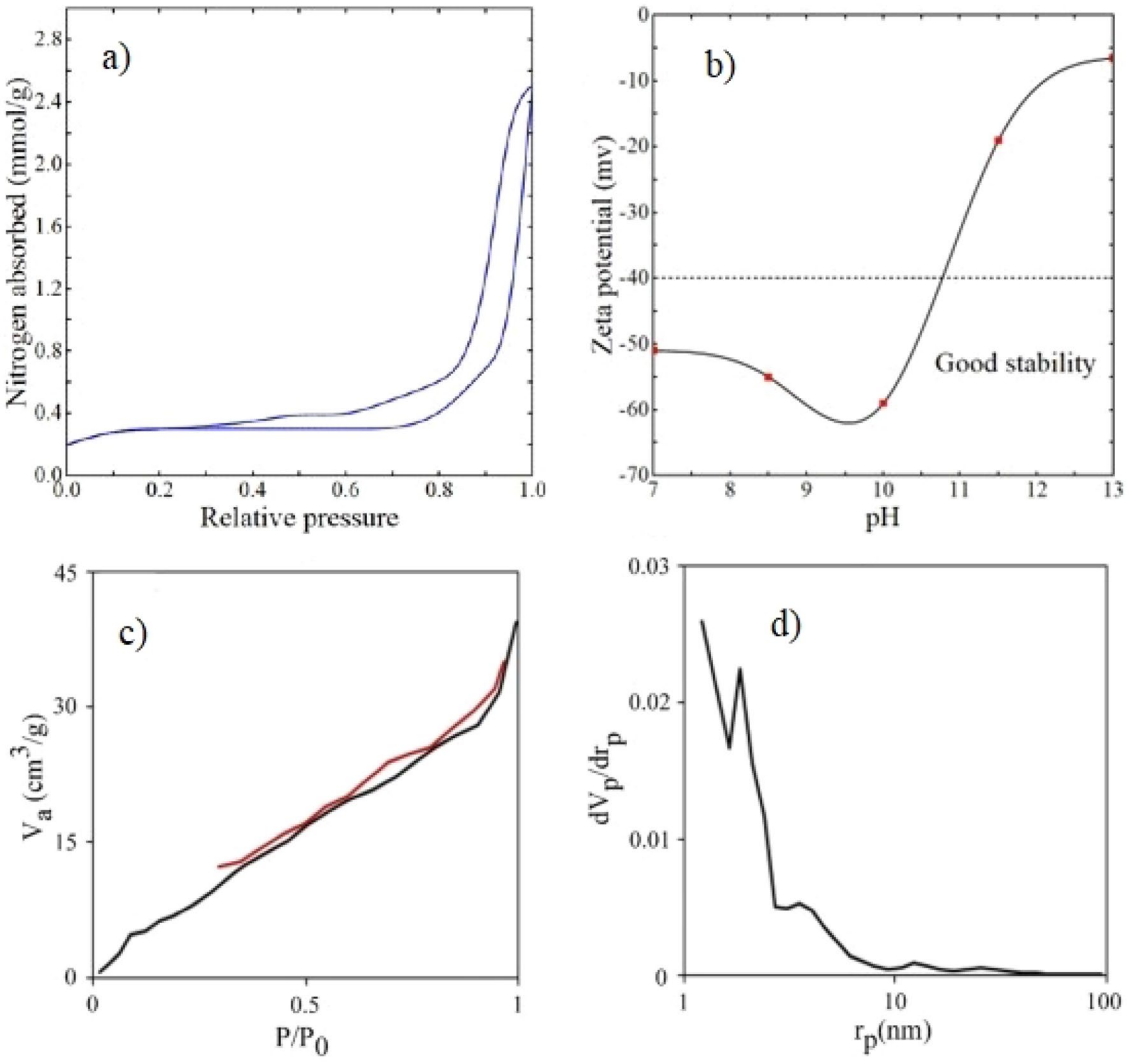
(**a**) Nitrogen adsorption–desorption isotherms of $\text{g} - {\text{C}}_{3}{\text{N}}_{4}$, (**b**) Average values of $\text{g} - {\text{C}}_{3}{\text{N}}_{4}$ zeta potential in aqueous suspensions at different pH, (**c**) nitrogen adsorption–desorption isotherms and (**d**) pore size distribution of the membrane.

The porosity of the g-C_3_N_4_ nanosheets was analysed using BET technique based on N_2_ adsorption–desorption isotherm as shown in Fig. 4a and Table S2. The g-C_3_N_4_ average pore size is less than 50 nm (9.04 nm), showing a mesoporous material according to the IUPAC classification. The specific surface area is 36.31 m^2^/g, significantly higher than the reported values in the literature for pure g-C_3_N_4_: 8.56 m^2^/g^40^ and 14.67 m^2^/g^41^. The elevated specific surface area of the g-C_3_N_4_ synthesised in this study may be related to the additional sonication step during the thermal polycondensation of melamine. The characteristic hysteresis isotherm of type IV in the P/P_0_ range of 0.4–1.0 can indicate a uniform pore size distribution^37^.

Zeta potential analysis was conducted at various pH levels at the room temperature to assess the surface charge and stability of g-C_3_N_4_ (Fig. 4b). The synthesised photocatalyst showed a relatively low stability at higher pH levels, as evidenced by its low zeta potential, and the maximum stability can be seen at a pH of 10. It was also reported that the emulsion of the g-C_3_N_4_ photocatalyst maintained its stability at neutral pH^42^. The detailed data of Zeta potential analysis can be found in Table S3 in the supplementary data.

### Membrane characterisation

**Figure 5:**
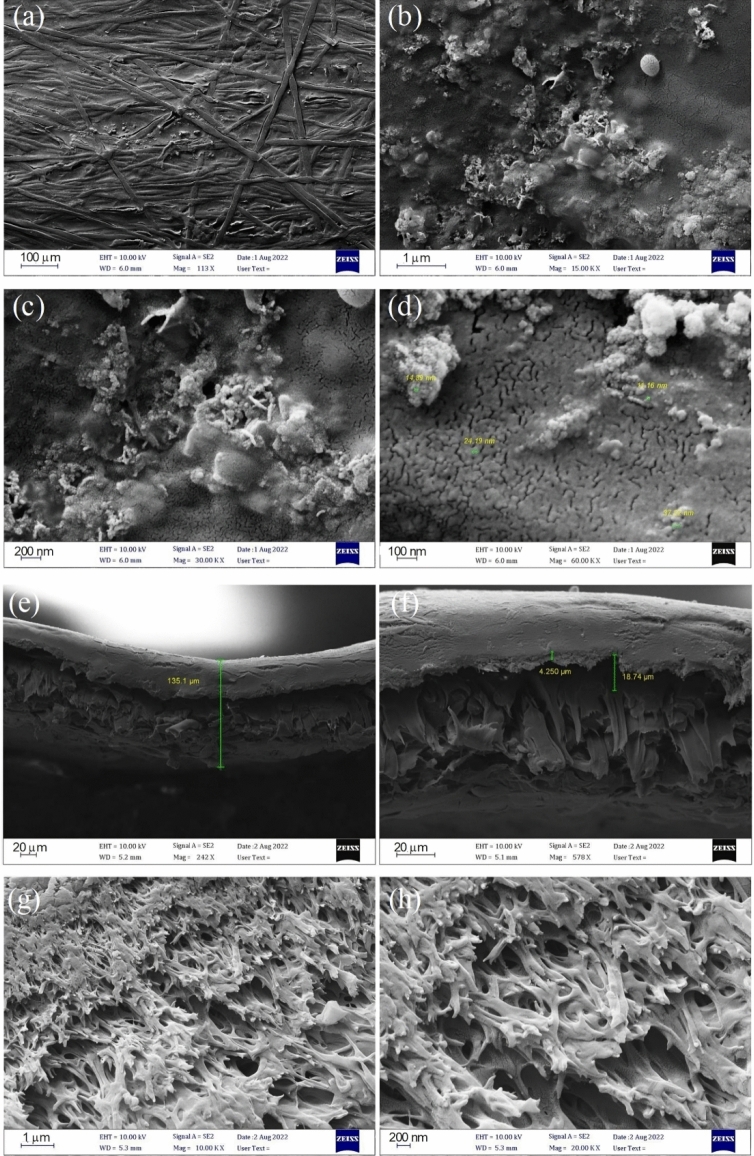
(**a**–**c**) FESEM images of the surface and (**d**–**h**) FESEM cross-section images of the membrane of membrane.

The membrane's porosity, pore size distribution, specific surface area, and total pore volume were determined using BET analysis and provided in Table S4. The membrane average pore diameter about 4.75 nm, placing it in the category of mesoporous materials^43^. Figure 4c displays N_2_ adsorption–desorption isotherm for the membrane, corresponding to type VI of the IUPAC classification. The isotherm shows that the membrane is composed of multiple layers of different pore sizes^44^. According to Fig. 4d, most of the pores have a diameter between 2.4 to 5.4 nm. The number of pores with a size larger than 5.4 nm decreases drastically. The membrane morphology evaluated by FESEM can be seen in Fig. 5a–c showing the groove like pores of the membrane.

Figure 5e–h displays the cross-section images of the membrane. The membrane displayed a characteristic asymmetrical shape, with sponge-like structures in the intermediate layers of the membrane and a dense skin layer on the top and bottom. Furthermore, it was clear that each membrane exhibited a uniformly porous interior structure. The results of the analysis of the membrane's contact angle are shown in Fig. S3. Higher membrane hydrophilicity results from a lower contact angle. These were determined by measuring the contact angle of a static water drop at 25 °C room temperature. The contact angle measurements showed that the membrane has a water contact angle of 0° (after three independent experiments), indicating the super hydrophilic property of this membrane.

**Figure 6:**
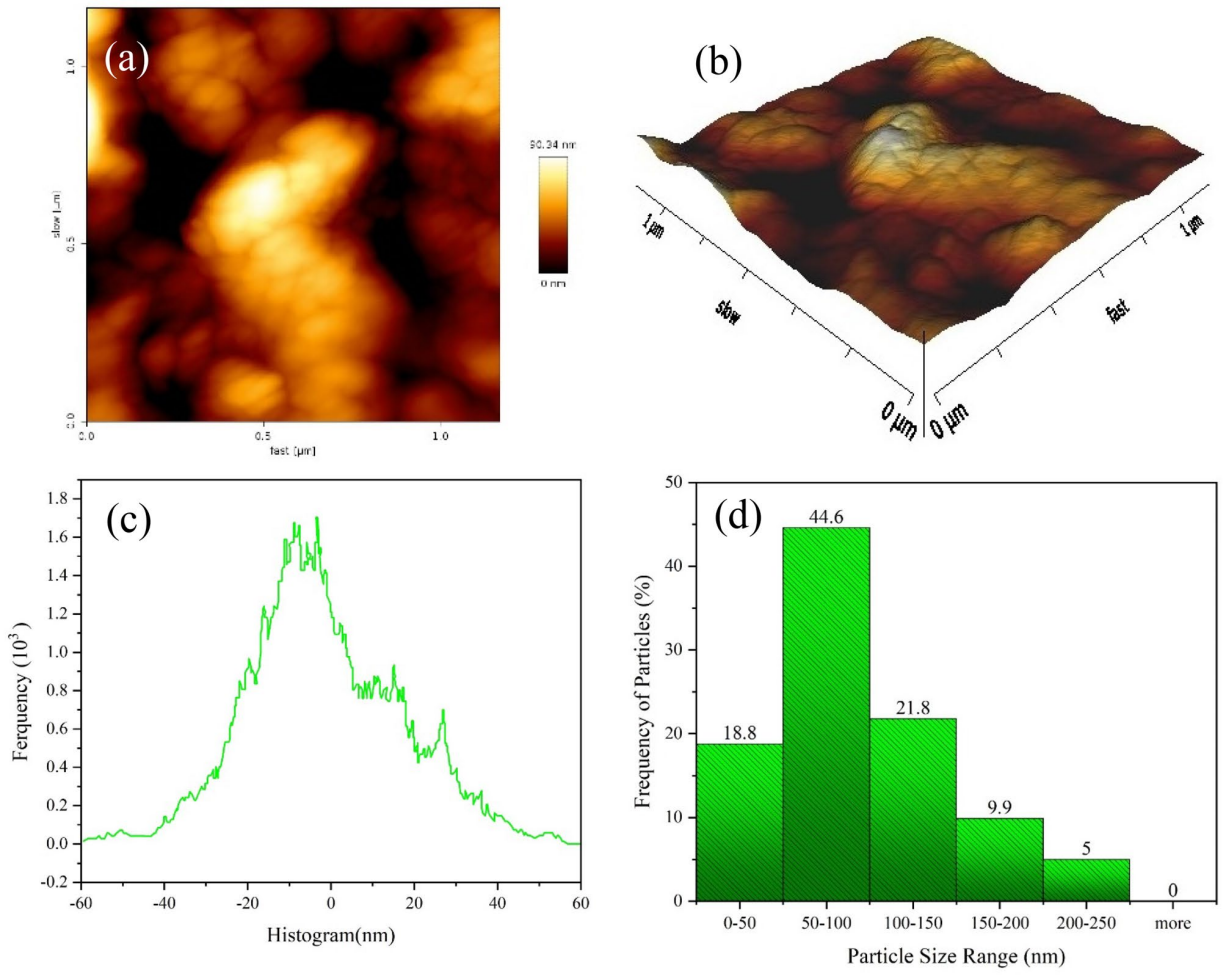
(**a**,**b**) AFM images of Membrane, (**c**) particle size distribution histogram for membrane (average of particle size = 95.6 nm) and (**d**) histograms of roughness for membrane (pick to valley roughness = 130.0 nm**).**

Figure 6a,b displays the membrane's 2 and 3D dimensional AFM images at scan sizes of 1.16 µm × 1.6µm. In order to precisely assess the roughness, Table S5 provides the mean distance (Rq) between peaks and valleys, the average roughness (Ra), and the difference (Rz) between high peaks and low valleys that were calculated from AFM analysis. The results, which show Ra = 16.23, Rq = 20.53, and Rz = 130.0 nm, imply that a smoother membrane surface (which is shown in Figs. 5 and 6a,b) reduces the severity of fouling and increases permeate flux because fewer foulants would be absorbed within the valleys and deposited on the membrane surface. Additionally, Fig. 6c displays the histograms of peak distribution and roughness and Fig. 6d displays Particle Size Distribution of membrane sample.

### Preliminary control testing

To assess the potential adsorption of TC on g-C_3_N_4_, the strategy of dark control test was followed. A 100 ml emulsion of 20 mg/L TC and 0.5 g/L g-C_3_N_4_ was prepared in a beaker. The beaker was then tightly covered with thick aluminium foil to ensure a dark environment and kept undisturbed for 24 h. The initial pH of the sample was about 6.6. TC concentration in the sample was recorded using UV–Vis spectroscopy, and just 2.2% of TC was removed through dark adsorption which can be attributed to the limited electrostatic interaction between TC and g-C_3_N_4_ at a pH of 6.6. The photocatalytic degradation of TC was tested with varying parameters of the irradiation time (60–120 min), initial pH (7–13), catalyst concentration (0.2–1 g/L), and initial TC concentration (10–30 mg/L). The reaction mixture was sampled, centrifuged, and analysed using UV–Vis spectroscopy as summarised in Table S6. The highest TC degradation of 63% was achieved in run number of 4, as determined by the Design-Expert software and based on the proposed operating conditions. The separability of the used membrane for TC was evaluated via 15 filtration cycles of 20 mg/L TC aqueous solution analysed by UV–Vis spectroscopy (Fig. S4 in supplementary information). The maximum TC removal achieved was 74.4%, mainly due to adsorption and size exclusion mechanisms. The molecular weight of TC is 480.9 g/mol, which is close to the membrane molecular weight cut-off (MWCO) of 400 Da can indirectly points to the size exclusion as a crucial role in removing TC from the aqueous solution^45,46^.

### PMR-based removal of TC

In this study, the performance of a split-type PMR in removal of TC from water was optimised using Design-Expert software based on CCD-based RSM optimization, in terms of five independent parameters of irradiation time, pH, catalyst dosage, TC initial concentration, and filtration iteration. This approach effectively minimises the number of experiments required, simplifies the identification of synergistic or antagonistic effects among factors, validates the obtained data, and quantifies the interactions between different factors. Based on the optimisation outcome, 50 tests were carried out and their results are tabulated in Tables S5. The maximum and minimum removal of TC using the split-type PMR were reported as 92% and 57%, respectively.

### ANOVA analysis

The results of ANOVA analysis for removal of TC from water are provided in Table 1 and Table S7. Following PMR-based removal of TC, regression models were developed using CCD method to determine the relationship between removal efficiency and five independent variables. The variables, namely irradiation time, pH, catalyst dosage, TC initial concentration, and filtration cycles, were noted as A, B, C, D, and E, respectively, in the proposed equation (Eq. 2). The equation includes a constant value, linear terms, and quadratic terms to show the individual effects of each parameter, as well as cross product terms to evaluate the interactive effects of parameters on the response. After removing insignificant terms, the regression model was obtained, and the results are presented in Table 1. In this model, positive and negative coefficients represent a synergistic and antagonistic effect between the variables, respectively. Table 1 can confirm that each parameter individually played a significant role in PMR-based TC removal. The interaction of pH with catalyst dosage, TC initial concentration, and filtration cycles showed significant effects on the model. The variance analysis in Table S7 demonstrates the suitability of the proposed models with the Model F-value of 205.52 which indicates its significance. However, the lack of model fit is not significant which indicates that the model is capable of calculating random errors for the experimental data^47^. The plots of Predicted-vs-actual, normal probability, residuals vs run number, and box-cox are shown in Fig. S5 of supplementary information. Actual values were determined experimentally and predicted values were provided by the RSM model (Fig. S5a). Predicted-vs-Actual plot indicates that the model is adequate, ensuring the acceptability of the predicted model since almost all the points in both the plots lie on or in the vicinity of the diagonal line. The normal probability distribution of residuals is shown in Fig. S5b which depicts a high degree of fitness due to a linear profile with a minimal error; hence, the errors are distributed normally. In this study, based on the ANOVA results for responses in Table S7, the obtained R^2^, R^2^_adj_, and R^2^_pred_ values for the removal of TC by split-type PMR are 0.98, 0.98, and 0.96, respectively. This indicates the adequacy of the suggested quadratic model. As observed, the adequate precision of the model is 55.83. The adequate precision measures the signal to noise ratio. A signal to noise ratio larger than 4 indicates that the model is able to navigate the design space^48^.

2 $${\text{Removal}}=-304.3991+(0.6700\times {\text{A}})+(39.9333\times {\text{B}})+(113.0416\times {\text{C}})+(9.5033\times {\text{D}})+(8.2833\times {\text{E}})+(-1.6666\times {\text{B}}\times {\text{C}})+(-0.0583\times {\text{B}}\times {\text{D}})+(0.2916\times {\text{B}}\times {\text{E}})+(-0.0029\times {\text{A}}^{2})+(-2.0166\times {\text{B}}^{2})+(-85.3125\times {\text{C}}^{2})+(-0.2015\times {\text{D}}^{2})+(-0.7875\times {\text{E}}^{2})$$

### Effect of irradiation time and pH

**Figure 7:**
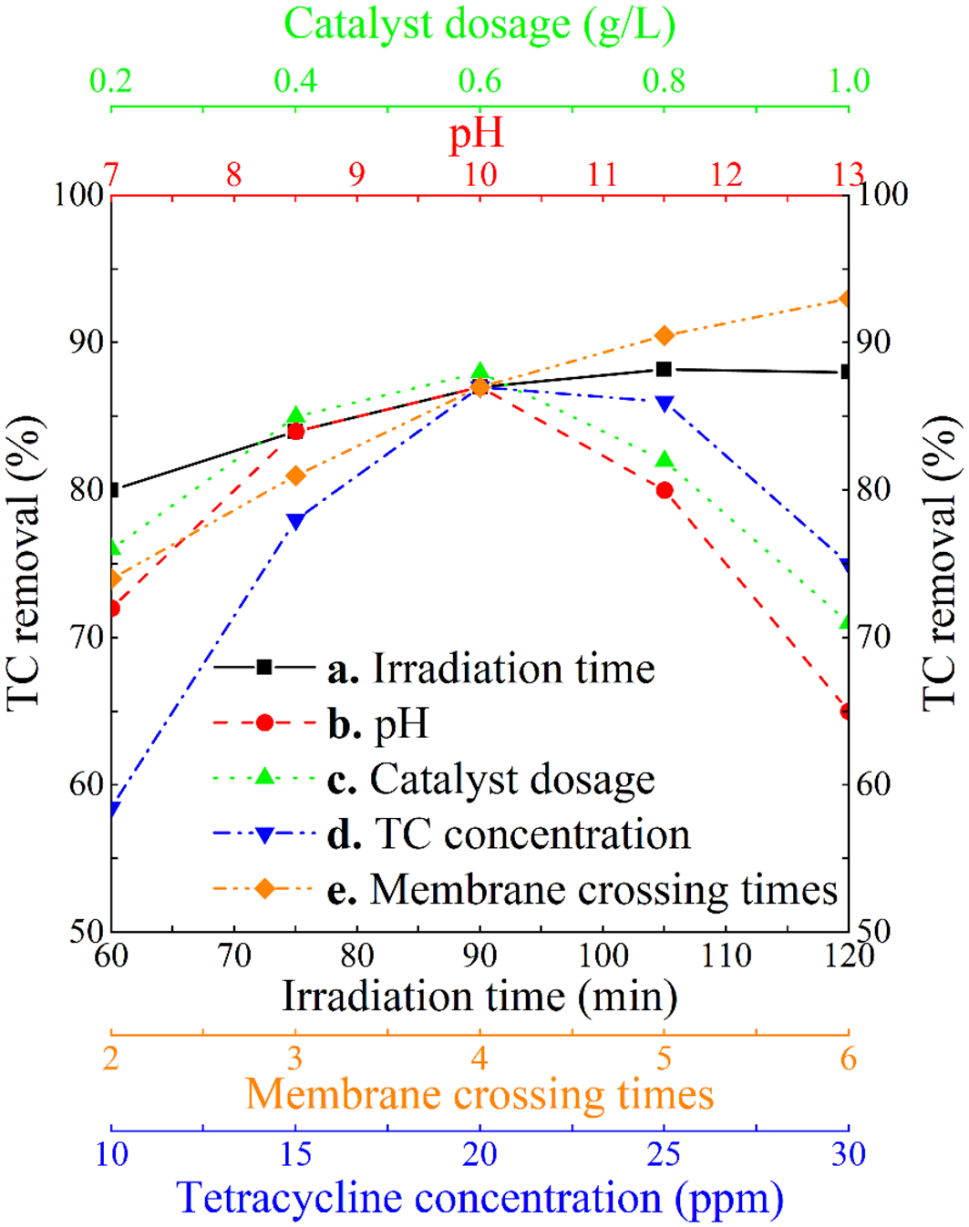
Effect of (**a**) irradiation time, (**b**) pH, (**c**) catalyst dosage, (**d**) TC initial concentration, and (**e**) number of passes through membrane on TC removal from aqueous solutions. Fixed parameters, if applicable, were irradiation time = 90 min, pH = 10, photocatalyst dosage = 0.6 g/L, TC initial concentration = 20 ppm, number of passes through membrane = 4.

Increasing the irradiation time initially enhanced TC removal due to the presence of empty active sites (Fig. 7a). However, as the irradiation duration increases and the active sites of the photocatalyst are being occupied, the impact of irradiation time diminishes^49,50^. The irradiation time has an optimal value, after which TC removal does not change. The maximum TC removal of 88.5% was achieved after 113.77 min at pH of 10, photocatalyst dosage of 0.6 g/L, TC initial concentration of 20 ppm, and 4 passes through the membrane. The impact of pH on TC removal using PMRs, as depicted in Fig. 7b, illustrates that TC removal initially increases with pH level, reaching its peak at 9.78. However, beyond that point, TC removal starts to decline with further increase in pH. The instability in photocatalyst reaction may be attributed to the change in surface electric charge of g-C_3_N_4_ nanosheets at varied pH level as also observed by Zeta potential analysis. g-C_3_N_4_ nanosheets had a negative electric charge over the pH range of 7–13, and particularly, at pH 10–13, it is lower in comparison to pH of 7–10. It is worth noting that, in the photocatalysis process, hydroxyl radicals play a crucial role^51^. At a higher pH, there are more hydroxide ions available within the solution, leading to increased production of hydroxyl radicals in the environment. However, as the pH continues to rise, hydroxide ions begin competing with TC molecules to occupy the photocatalyst active sites which negatively affects the photocatalytic removal of TC from aqueous solutions^52^. Overall, the maximum TC removal of 87% was achieved at specific operating conditions: pH level of 9.78, irradiation time of 90 min, photocatalyst dosage of 0.6 g/L, TC initial concentration of 20 ppm, and after 4 filtration cycles. As seen in Fig. 6a, the degradation efficiency of TC increases as the irradiation time increases from 60 to 105 min due to the enhanced interactions between TC and the photocatalyst, leading to the attack of hydroxyl radicals on TC and consequently degradation increase. However, when the irradiation time increased to 110min, degradation efficiency remains constant. This is likely due to the decrease in the number of active sites available for photocatalytic interactions. Beyond 110min, the degradation efficiency decreases as all available active sites become saturated, leading to no further increase and negative effect on degradation efficiency.

### Effect of catalyst dosage

Figure 7c presents the impact of catalyst dosage on the PMR-based removal of TC. In essence, augmenting the photocatalyst concentration can enhance surface area availability for photon absorption, thereby accelerating oxidation reactions^53^. However, a higher chemical density of photocatalyst in the reactor can also lead to the development of a turbid suspension, diminishing its transparency and photo penetration depth^54^. Furthermore, adding photocatalyst in a turbid suspension may result in photocatalyst aggregation and limit photocatalyst activities. Consequently, initially, raising the photocatalyst concentration linearly elevates photocatalytic removal rate; however, surpassing the optimal concentration of photocatalyst may not only fail to further increase TC removal but actually diminish it^55^. It is evident that the optimal photocatalyst loading should be determined as a very crucial parameter controlling photocatalyst activities from operational and economic viewpoints. The maximum TC removal of 87.5% was recorded at the photocatalyst dosage of 0.56 g/L, irradiation time of 90 min, pH of 10, TC initial concentration of 20ppm, and after 4 filtration iterations. This study discloses that increasing the amount of photocatalyst from 0.2 up to 0.6 g/L can effectively elevate the rate of degradation. However, at higher concentrations, beyond 0.6 g/L, light scattering can occur, reducing the transparency of the solution and decreasing photon access to the photocatalyst surface. Another reason for the efficiency reduction is the accumulation of catalysts in a clustered form, leading to a decrease in photon adsorption in the photocatalytic process. Therefore, the efficiency of photocatalytic degradation decreases with an increase in the amount of photocatalyst (> 6 g/L).

### Effect of TC initial concentration

Figure 7d showcases the effects of pollutant initial concentration on the overall performance of PMR system. Within a certain range, elevating the pollutant primary concentration enhances the collision rate and oxidation reaction, leading to a better degradation up to an optimal dosage after which a relatively lower degradation rate is probable^22^ due to increased emulsion turbidity and limited light absorbency. TC molecules at high concentrations may occupy the active sites of the photocatalyst, exerting a negative influence on the photocatalytic degradation process^13,56^. The maximum removal of TC (88.5%) was recorded at a TC concentration of 22.16 ppm, with an irradiation time of 90 min, pH of 10, photocatalyst dosage of 0.6 g/L, and after four membrane filtration cycles. As the initial concentration of TC increases, the electron–hole pair ratio decreases, and active sites on the surface of the photocatalyst become saturated by TC molecules. This leads to less light entering the photocatalytic degradation system, and the efficiency of TC photocatalytic degradation decreases. Another reason may be the turbidity of the solution in which the photocatalytic reactions take place. The solution becomes cloudy, making it difficult for light to pass through, which reduces the amount of light irradiation on the photocatalyst surface and consequently reduces photocatalytic degradation efficiency.

#### Effect of membrane filtration cycle

Figure 7e provides some insight into the impact of membrane separation iterations on the TC removal using the developed PMR system. It is evident that more cycles of membrane filtration can result in higher amount of captured TC mainly due to size exclusion as evidenced based in the outcome of BET analysis. The maximum TC removal rate of 94.8% was achieved following an irradiation time of 90 min, at a pH level of 10, with a photocatalyst dosage of 0.6 g/L, an initial TC concentration of 20 ppm, and a total of six passes through the membrane.

#### Optimization of process operational parameters

The operating conditions yielding maximum PMR-based TC removal were determined based a CCD-based RSM optimisation carried out by Design-Expert software. The optimal range of parameters considered are provided in Table S8, leading to a set of conditions exhibiting greater desirability and feasibility (Table S9). To ensure accuracy, reliability, and reproducibility, the optimal points were experimentally tested three times. The results presented in Table S9 highlight a maximum degradation of 94.8%, closely aligning with the predicted software value of 96.2%. The concordance between the predicted and experimental outcomes underscores the model's reliability and its ability to accurately anticipate the maximum degradation of TC. Furthermore, the photocatalytic performance of TC photodegradation in this study has been systematically compared with pertinent literature Table 2.

**Table 2:** Comparison of TC photocatalytic-degradation efficiency of this work with other visible-light-driven catalytic systems from recent literatures.

TC Con. (ppm)	Photocatalyst/membrane	Light source	Irradiation time (min)	Removal of TC (%)	Ref
40	SnO_2_-modified-g-C_3_N_4_	Three 50 W fluorescent lamps	120	90.29	^57^
10	Au-TiO2/pDA-PVDF	300W xenon	120	92	^58^
10	cobalt-doped ZnTiO_3_/Ti_3_C2Tx MXene	300 W xenon	1440	~ 80	^59^
20	MnFe_2_O_4_/BiOI	500 W xenon	200	83.04	^60^
0.1	ZnIn2S4/PVDF	150W halogen	2160	92	^61^
30	Type-1 α-Fe_2_O_3_/TiO_2_	500 W halogen	120	97.5	^62^
20	Bi_2_WO_6_-CeO_2_/PVDF MoO_3_/g-C_3_N_4_ Z-scheme	Visible light	200	82	^63^
20	TiO2-BiOBr-NCQDs/PVDF	300W xenon	120	77	^64^
20	AgBr nanoparticles decorated 2D/2D GO/Bi_2_WO_6_	350 W xenon	60	84	^65^
20	AgI/BiVO_4_ p–n junction	500 W xenon	100	~ 85	^66^
5	Au-TiO2/PVDF	300W xenon	120	75	^67^
10	AgBr–TiO_2_-Palygorskite	300W xenon	90	90	^68^
20	Bi2WO6-CeO2/PVDF	Visible light	200	82	^69^
22.16	g-C_3_N_4_ membrane reactor	300W xenon	143.776	94.8%	This work

#### Photocatalyst reusability and mechanism

**Figure 8:**
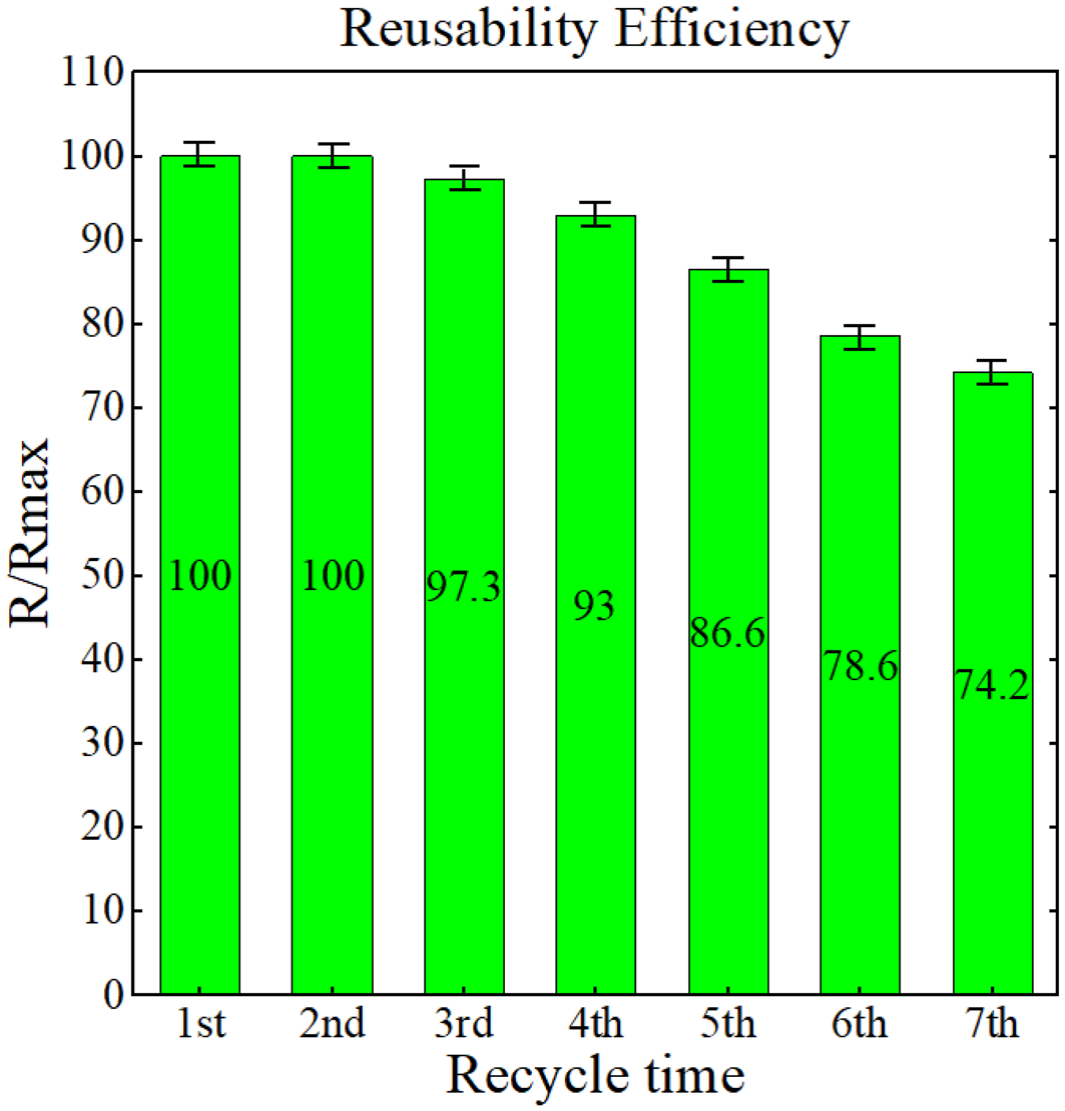
Photocatalyst removal efficiency in continuous cycles at optimum conditions.

**Figure 9:**
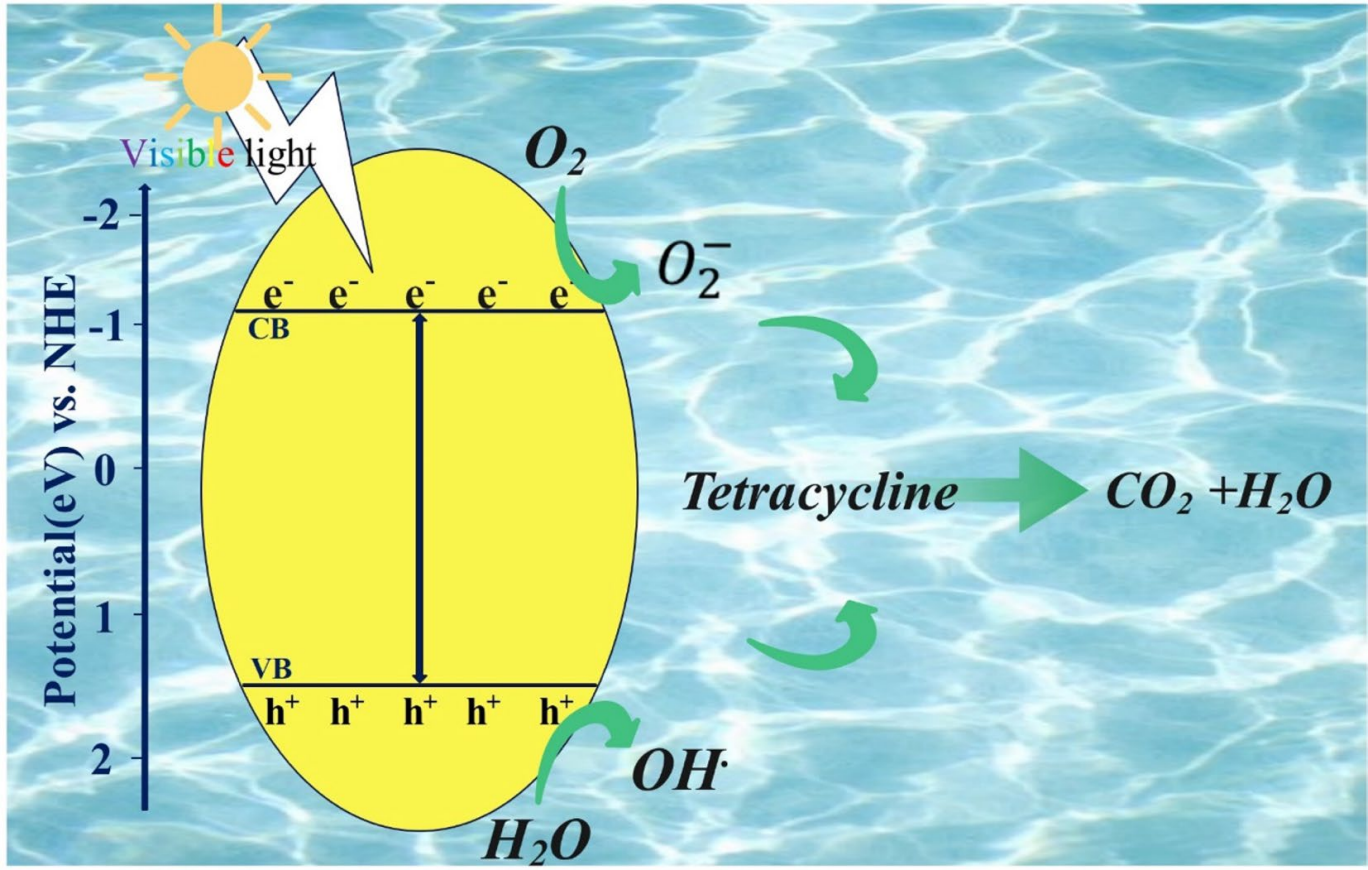
Schematic diagram for the possible g-C_3_N_4_ photodegradation mechanisms under visible-light irradiation.

The reusability of the synthesised photocatalyst in TC removal was analysed at optimal operating conditions (irradiation time of 113.77 min, pH level of 9.78, photocatalyst dosage of 0.56 g/L and TC concentration of 22.16 ppm) as an important marketability index as shown in Fig. 8. In brief, in each cycle, the used materials were recovered as follows. The photocatalyst was initially separated using centrifugation (at 7500 rpm for 7 min), then washed twice with 50 mL of ethanol (15 min magnetic stirring plus 6 min probe sonication at 100 watts), and followed by a complete DDW washing. The clean photocatalyst was oven-dried at 70 °C for 24 h. In order to investigate the economic justification of the g-C_3_N_4_ photocatalyst for TC removal from aqueous solutions^70–72^. After seven cycles, the photocatalyst's TC removal efficiency, as shown in Fig. 8, reached 74.2%. It can be mainly attributed to two primary factors of blockage and degradation of active sites. The superficial active sites of the photocatalyst may become partially blocked by remaining TC molecules and reaction products, interrupting the photocatalytic interaction. Cyclic photocatalysis can also lead to the degradation or alteration of some active sites. Surface fouling, catalyst aging, and exposure to reactive species may also contribute to the active sites’ deterioration, effectively diminishing their catalytic activities. These clues can highlight the importance of understanding the long-term challenges of photocatalysis. It also necessitates exploration of strategies for photocatalyst regeneration and optimisation to mitigate the decline in their removal efficiency^73^. The most crucial issue with fouling, degradation, or loss of photocatalytic activity over seven cycles is accumulation of TC molecules which may partially block the superficial active sites of the photocatalyst, impeding photocatalytic interactions. To mitigate the issues associated with the decline in photocatalyst efficiency after multiple cycles, it is required to improve the regeneration method to restore the photocatalyst's active sites after they have become partially blocked by TC molecules and reaction products over several cycles without causing significant damage or alteration to its structure. In addition, modification of the surface of the photocatalyst to make it less prone to blockage by TC molecules and reaction by-products can greatly contribute to addressing this challenge. According to previous works, ${{\text{O}}}_{2}^{-}$^74–77^ and h^+^^74,78^, respectively, plays a key role in the g-C_3_N_4_ photodegradation of TC. The potential g-C_3_N_4_ for TC degradation pathways are shown in Fig. 9 based on the reactive species mentioned above. Upon irradiation of visible light on the g-C_3_N_4_ photocatalyst, electrons and holes generate in the conduction and valence bands, respectively. As a result of the produced electrons and holes, TC is degraded down into CO_2_ and H_2_O by reduction and oxidation processes with oxygen and water, respectively.

It is also worth mentioning that the cost analysis, scaling viewpoints, and commercialisation aspects are all outside of the scope of this paper since the finalisation of scaling up/piloting is an imperative step before any valid cost analysis. However, cost analysis and feasibility study are recommended as topics for future research to pinpoint this technique. This study also does not discuss the environmental impact and sustainability issues of the photocatalyst and membrane used in the PMR system. It is crucial as topic for future research to assess the lifecycle impact of their synthesis, disposal, and potential release of harmful substances/by-products. Despite the promising performance, addressing these environmental impacts is vital in assessing the long-term sustainability of PMR technologies in practical settings.

## Conclusions

This study investigated the degradation of tetracycline from water using a lab-scale photocatalytic membrane reactor (PMR) with a suspended graphitic carbon nitride (g-C3N4) photocatalyst and a layered polymeric polyester/polysulfone/polyamide membrane. A range of operating conditions were explored, and optimal parameters of irradiation time: 113.77min, pH: 9.78, photocatalyst dosage: 0.56g/L, tetracycline initial concentration: 22.16 mg/L, and 6 membrane passes resulted in a tetracycline removal efficiency of about 95%. The proposed hybrid approach, compared to individual photocatalysis and membrane processes, was confirmed to have about 32% and 20% higher removal efficiency, respectively. The designed PMR showed reasonable photocatalyst reusability, reaching 74% of maximum removal efficiency after seven cycles. Overall, the study's outcomes support the efficacy of the proposed PMR for tetracycline removal, offering a sustainable water treatment solution with a feasible hybrid photocatalyst-membrane process.

### Supplementary Information

Supplementary Information.

## Data Availability

The datasets employed or examined in the present study can be obtained from the corresponding authors upon reasonable request.
