# Supplementary material for: Tetracycline removal from wastewater via g-C3N4 loaded RSM-CCD-optimised hybrid photocatalytic membrane reactor
Source: Sci Rep. 2024 Jan 12;14:1163. doi: 10.1038/s41598-024-51847-5 (PMC10786873; doi:10.1038/s41598-024-51847-5)
Supplement: Supplementary file 1 — Supplementary Information. [file 41598_2024_51847_MOESM1_ESM.docx]

**SUPPORTING INFORMATION**

Tetracycline Removal from Wastewater *via* g-C_3_N_4_ Loaded RSM-CCD-Optimised Hybrid Photocatalytic Membrane Reactor

Milad Esfandiaribayat^1^, Mojtaba Binazadeh^1,2*^, Samad Sabbaghi^3^, Milad Mohammadi^3^

Samaneh Ghaedi^4^, Hamid Rajabi^5,*^

*^1^Department of Chemical Engineering, School of Chemical and Petroleum Engineering, Shiraz University, Shiraz, Iran.*

*^2^Department of Civil and Environmental Engineering, University of Alberta, Alberta T6G 2W2, Canada.*

*^3^Department of Nano-Chemical Engineering, Faculty of Advanced Technologies, Shiraz University, Shiraz, Iran.*

*^4^Department of Mechanical, Aerospace and Civil Engineering, School of Engineering, the University of Manchester, Manchester M13 9PL, UK.*

*^d^ Department of Civil and Environmental Engineering, School of Engineering, University of Liverpool, Liverpool L69 3GH, UK.*

* Corresponding Authors:

E-mail: binazadeh@shirazu.ac.ir, binazade@ualberta.ca

Address: (1) Department of Chemical Engineering, School of Chemical and Petroleum Engineering, Shiraz University, Shiraz, Iran. (2) Department of Civil and Environmental Engineering, University of Alberta, Alberta T6G 2W2, Canada

Email: hamid.rajabi@liverpool.ac.uk

Address: Harrison Hughes Building, School of Engineering, University of Liverpool, Liverpool L69 3GH, UK.

**Number of Pages: 7**

**Number of Tables: 9**

**Number of Figures: 3**

**Contents:**

**Table S1.** The main properties of commercial membrane.

**Table S2.** Zeta potential analysis data at different pH.

**Table S3.** $\text{g-}\text{C}_{\mathbf{3}}\text{N}_{\mathbf{4}}$ BET analysis results.

**Table S4.** Membrane BET analysis results.

**Table S5.** AFM surface roughness parameters for Membrane

**Table S6.** Experimental design matrix and the final results of tetracycline removals.

**Table S7.** Analysis of variance (ANOVA) results for responses.

**Table S8.** Experimental conditions set in Design-Expert for optimization.

**Table S9.** Predicted and experimental values of the studied responses at optimum conditions.

**Fig. S1.** Real photo of PMR system.

**Fig. S2.** Calibration curve of tetracycline in double distilled water.

**Fig. S3.** Contact angle image of membrane.

**Fig. S4.** Tetracycline rejection with membrane separation process.

**Fig. S5.** a. Predicted vs actual, b. normal probability distribution, c. residuals vs run number, and d. Box-Cox plots for degradation of TC via photocatalytic membrane reactor.


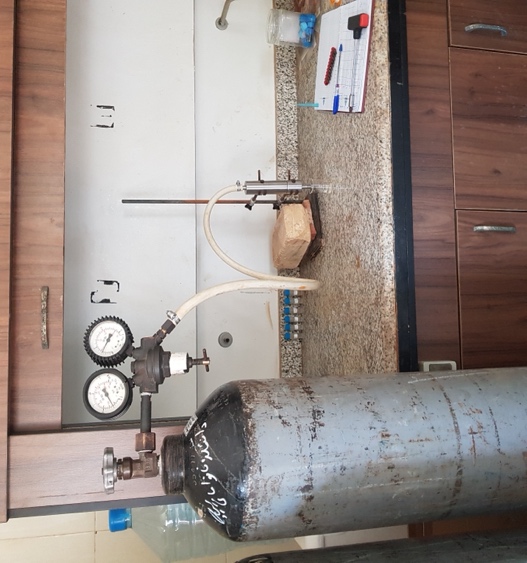

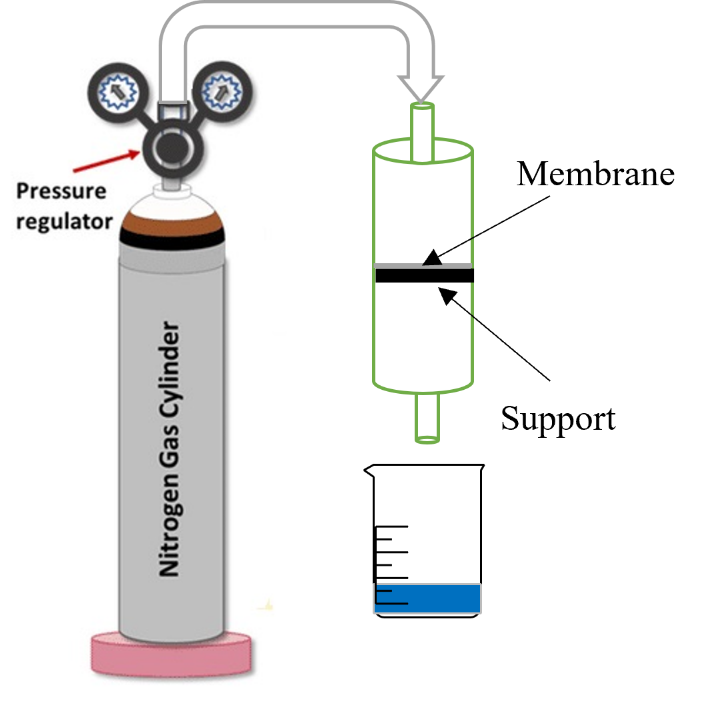


**Fig. S1.** Real photo of PMR system.


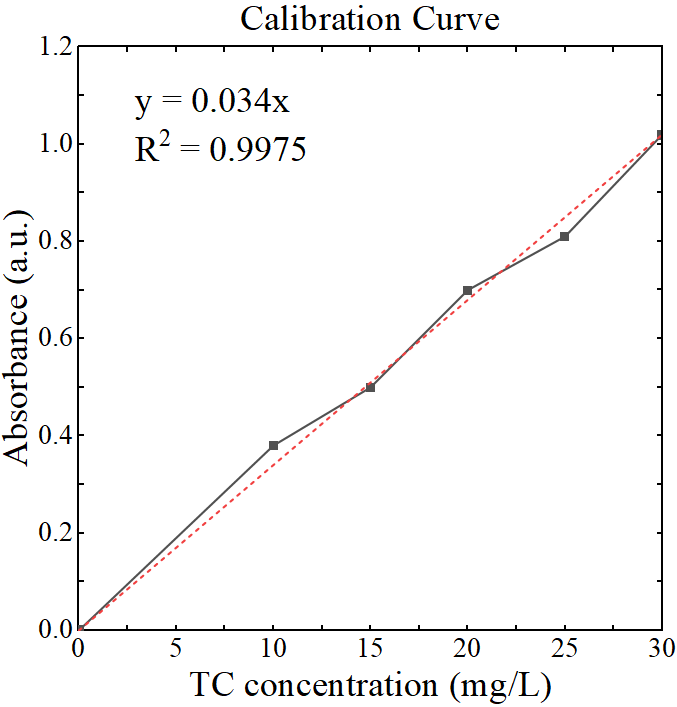


**Fig. S2.** Calibration curve of tetracycline in double distilled water.


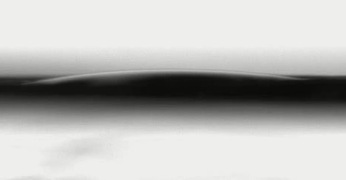


**Fig. S3.** Contact angle image of membrane


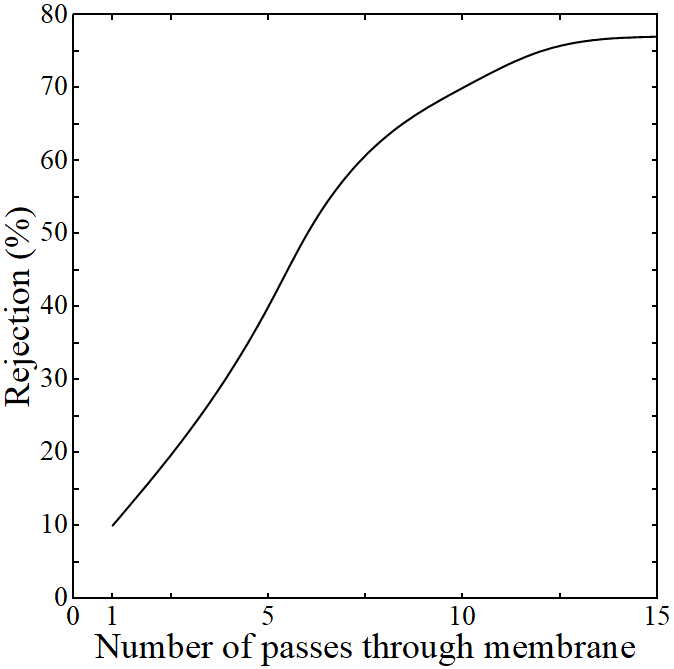


**Fig. S4.** Tetracycline rejection with membrane separation process.


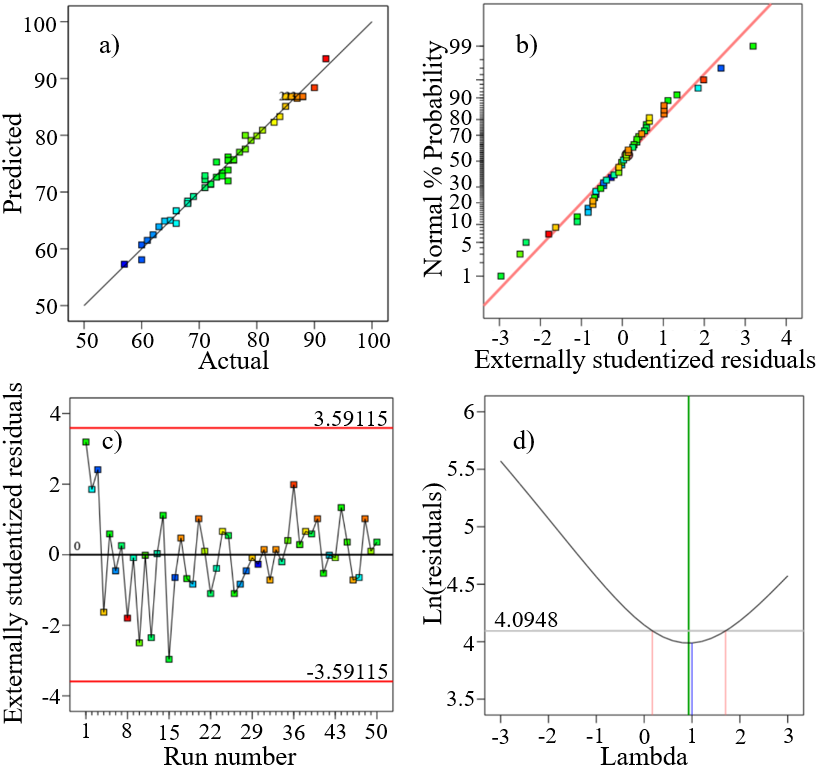


**Fig. S5. a.** Predicted vs actual, **b.** normal probability distribution, **c.** residuals vs run number, and **d.** Box-Cox plots for degradation of TC *via* photocatalytic membrane reactor.

Table S1. The main properties of commercial membrane.

| **Parameter** | **Range/type** |
| --- | --- |
| Material | Polyester/Polysulfone/Polyamide |
| Operating pH | 3-10 |
| TMP (bar) | 2-13 |
| MWCO (Da) | 400 |
| Geometry | Flat sheet |

Table S2. $\text{g-}\text{C}_{\mathbf{3}}\text{N}_{\mathbf{4}}$ BET analysis results.

| **Parameter** | **Value** |
| --- | --- |
| BET Surface Area (m^2^/g) | 36.31 |
| Langmuir Specific Area (m^2^/g) | 44.69 |
| Pore Size (nm) | 9.04 |
| Pore Diameter (nm) | 13.29 |
| Pore Volume (cm^3^/g) | 0.19 |

**Table S3.** Zeta potential analysis data at different pH.

| **pH** | **Zeta potential values (mv)** | | | | **Average zeta potential value (mv)** |
| --- | --- | --- | --- | --- | --- |
|  | **Test1** | **Test2** | **Test3** | **Test4** |  |
| 7 | -49.6 | -53.6 | -54.2 | -52.7 | -55.5 |
| 8.5 | -53.0 | -52.7 | -55.7 | -57.1 | -54.6 |
| 10 | -58.1 | -59.7 | -58.1 | -60.6 | -59.1 |
| 11.5 | -19.8 | -18.0 | -19.5 | -17.5 | -18.7 |
| 13 | -9.7 | -4.0 | -5.8 | -7.7 | -6.7 |

Table S4. $\text{Membrane}$ BET analysis results.

| **Parameter** | **value** |
| --- | --- |
| BET surface area (m^2^/g) | 50.32 |
| Langmuir specific area (m^2^/g) | 676.78 |
| Average pore diameter (nm) | 4.75 |
| Total pore volume (cm^3^/g) | 0.05 |

**Table S5.** AFM surface roughness parameters for Membrane

| Membrane | Ra(nm) | Rq(nm) | Rz(nm) | Average value |
| --- | --- | --- | --- | --- |
| PES/PS/PA | 16.23 | 20.53 | 130 | 4.043 pm |

Table S6. Experimental design matrix and the final results of tetracycline removals.

| **Run** | **Irradiation time**  **(min)** | **pH** | **Cat. dosage**  **(g/L)** | **TC initial concentration**  **(mg/L)** | **# of Passes through membrane** | **Photocatalysis**  **removal**  **(%)** | **Total removal (%)** |
| --- | --- | --- | --- | --- | --- | --- | --- |
| 1 | 75 | 8.5 | 0.8 | 25 | 3 | 51 | 75 |
| 2 | 90 | 13 | 0.6 | 20 | 4 | 41 | 66 |
| 3 | 90 | 10 | 0.6 | 10 | 4 | 50 | 60 |
| 4 | 90 | 10 | 0.6 | 20 | 4 | 63 | 85 |
| 5 | 75 | 8.5 | 0.4 | 25 | 3 | 54 | 74 |
| 6 | 75 | 8.5 | 0.8 | 15 | 3 | 46 | 62 |
| 7 | 90 | 10 | 1 | 20 | 4 | 56 | 71 |
| 8 | 90 | 10 | 0.6 | 20 | 6 | 61 | 92 |
| 9 | 105 | 8.5 | 0.4 | 15 | 3 | 49 | 68 |
| 10 | 60 | 10 | 0.6 | 20 | 4 | 55 | 78 |
| 11 | 105 | 8.5 | 0.4 | 15 | 5 | 48 | 77 |
| 12 | 90 | 7 | 0.6 | 20 | 4 | 49 | 71 |
| 13 | 75 | 11.5 | 0.8 | 15 | 5 | 39 | 68 |
| 14 | 75 | 8.5 | 0.4 | 15 | 5 | 45 | 74 |
| 15 | 90 | 10 | 0.6 | 30 | 4 | 46 | 73 |
| 16 | 75 | 11.5 | 0.4 | 15 | 3 | 42 | 60 |
| 17 | 105 | 8.5 | 0.4 | 25 | 5 | 55 | 87 |
| 18 | 90 | 10 | 0.2 | 25 | 4 | 44 | 75 |
| 19 | 105 | 11.5 | 0.4 | 15 | 3 | 48 | 64 |
| 20 | 75 | 10 | 0.6 | 20 | 4 | 61 | 88 |
| 21 | 75 | 8.5 | 0.8 | 25 | 5 | 50 | 81 |
| 22 | 105 | 11.5 | 0.8 | 15 | 5 | 44 | 71 |
| 23 | 75 | 11.5 | 0.4 | 25 | 3 | 48 | 68 |
| 24 | 75 | 8.5 | 0.4 | 25 | 5 | 53 | 83 |
| 25 | 75 | 8.5 | 0.8 | 15 | 5 | 45 | 72 |
| 26 | 105 | 8.5 | 0.8 | 25 | 3 | 54 | 75 |
| 27 | 75 | 8.5 | 0.4 | 15 | 3 | 46 | 63 |
| 28 | 105 | 11.5 | 0.8 | 15 | 3 | 44 | 61 |
| 29 | 105 | 11.5 | 0.8 | 25 | 5 | 54 | 85 |
| 30 | 75 | 11.5 | 0.8 | 15 | 3 | 39 | 57 |
| 31 | 90 | 10 | 0.6 | 20 | 4 | 61 | 87 |
| 32 | 90 | 10 | 0.6 | 20 | 4 | 60 | 86 |
| 33 | 90 | 10 | 0.6 | 20 | 4 | 60 | 87 |
| 34 | 105 | 11.5 | 0.8 | 25 | 3 | 48 | 69 |
| 35 | 105 | 8.5 | 0.4 | 25 | 3 | 55 | 78 |
| 36 | 120 | 10 | 0.6 | 20 | 4 | 62 | 90 |
| 37 | 75 | 11.5 | 0.8 | 25 | 5 | 43 | 76 |
| 38 | 105 | 11.5 | 0.4 | 25 | 5 | 54 | 84 |
| 39 | 75 | 11.5 | 0.4 | 15 | 5 | 42 | 72 |
| 40 | 90 | 10 | 0.6 | 20 | 4 | 61 | 88 |
| 41 | 105 | 11.5 | 0.4 | 15 | 5 | 48 | 75 |
| 42 | 75 | 11.5 | 0.8 | 25 | 3 | 43 | 65 |
| 43 | 75 | 11.5 | 0.4 | 25 | 5 | 48 | 79 |
| 44 | 90 | 10 | 0.6 | 20 | 2 | 61 | 75 |
| 45 | 105 | 8.5 | 0.8 | 15 | 5 | 49 | 76 |
| 46 | 90 | 10 | 0.6 | 20 | 4 | 61 | 86 |
| 47 | 105 | 8.5 | 0.8 | 15 | 3 | 61 | 66 |
| 48 | 90 | 10 | 0.6 | 20 | 4 | 60 | 88 |
| 49 | 105 | 11.5 | 0.8 | 25 | 5 | 48 | 80 |
| 50 | 105 | 11.5 | 0.4 | 25 | 3 | 54 | 73 |

Table S7. Analysis of variance (ANOVA) results for responses.

| $\text{R}^{\text{2}}$ | Adjusted $\text{R}^{\text{2}}$ | Predicted $\text{R}^{\text{2}}$ | Adequate precision | STD. DEV. | Mean | C.V. % |
| --- | --- | --- | --- | --- | --- | --- |
| 0.98 | 0.98 | 0.96 | 55.83 | 1.22 | 75.28 | 1.63 |

Table S8. Experimental conditions set in Design-Expert for optimization.

| **Parameter** | **Range/Goal** |
| --- | --- |
| Irradiation time (min) | 60-120 |
| pH | 7-13 |
| Catalyst dosage (g/L) | 0.2-1 |
| TC initial concentration | 10-30 |
| # of passes through membrane | 2-6 |
| Removal (%) | maximum |

Table S9. Predicted and experimental values of the studied responses at optimum conditions.

| **Irradiation time (min)** | **pH** | **Cat. dosage (g/L)** | **TC initial conc. (mg/L)** | **# of passes through membrane** | **Removal (%)** | | | |
| --- | --- | --- | --- | --- | --- | --- | --- | --- |
|  |  |  |  |  | **Predicted** | **Test 1** | **Test 2** | **Test 3** |
| 113.77 | 9.78 | 0.56 | 22.16 | 6 | 96.2 | 95 | 94.6 | 94.8 |
